# Microstructure and Corrosion Properties of AlCrFeCoNi High-Entropy Alloy Coatings Prepared by HVAF and HVOF

**DOI:** 10.1007/s11666-021-01255-2

**Published:** 2021-09-09

**Authors:** Martin Löbel, Thomas Lindner, Thomas Mehner, Lisa-Marie Rymer, Stefan Björklund, Shrikant Joshi, Thomas Lampke

**Affiliations:** 1 Materials and Surface Engineering Group, Institute of Materials Science and Engineering, Chemnitz University of Technology, 09107 Chemnitz, Germany; 2 Department of Engineering Science, University West, 46132 Trollhättan, Sweden

**Keywords:** coating, corrosion, HEA, HVOF, HVAF, high-entropy alloy, microstructure

## Abstract

High-entropy alloys (HEAs) represent an innovative development approach for new alloy systems. These materials have been found to yield promising properties, such as high strength in combination with sufficient ductility as well as high wear and corrosion resistance. Especially for alloys with a body-centered cubic (bcc) structure, advantageous surface properties have been revealed. However, typical HEA systems contain high contents of expensive or scarce elements. Consequently, applying them as coatings where their use is limited to the surface represents an exciting pathway enabling economical exploitation of their superior properties. Nevertheless, processing conditions strongly influence the resulting microstructure and phase formation, which in turn has a considerable effect on the functional properties of HEAs. In the presented study, microstructural differences between high-velocity oxygen fuel (HVOF) and high-velocity air fuel (HVAF) sprayed coatings of the alloy AlCrFeCoNi are investigated. A metastable bcc structure is formed in both coating processes. Precipitation reactions are suppressed by the rapid solidification during atomization and by the relatively low thermal input during spraying. The coating resistance to corrosive media was investigated in detail, and an improved passivation behavior was observed in the HVAF coatings.

## Introduction

The multi-principal group of high-entropy alloys (HEAs) has gained extensive research interest. Their properties mainly stem from the ability to form simple solid solutions, preferably with body-centered cubic (bcc) or face-centered cubic (fcc) structure. Despite their complex composition, usually comprised of at least four elements with approximately equimolar composition, the formation of complex and brittle phases can be suppressed. Furthermore, improved properties could be achieved by minor elemental additions of non-metallic elements (Ref 1). Extensive investigations revealed an interesting combination of properties, e.g., high hardness and strength in combination with high ductility, which make these alloys promising for structural applications. Additionally, a high wear resistance under various loads and conditions was determined (Ref 2, 3). These properties also make HEAs feasible for coating applications, where the required quantities of these materials can be significantly reduced by limiting them to the surface. Thermally sprayed HEA coatings have been produced utilizing various feedstock production routes and coating processes (Ref 4, 5). The best results in terms of homogeneity and defect reduction have been achieved by processing gas-atomized feedstock using high-kinetic energy thermal spray processes (Ref 6, 7). One such industrially well-established process is high-velocity oxygen fuel (HVOF) thermal spraying, which enables the deposition of coatings with low porosity and oxide content. A further advancement of this process is high-velocity air fuel (HVAF) thermal spraying, where the combustion temperature is reduced by utilizing air in place of oxygen for fuel combustion. In this way, the formation of oxides and thermal degradation of the feedstock, e.g., metals or cemented carbides, can be avoided (Ref 8, 9). This process had not yet been considered for HEAs.

The most important base alloy system of HEAs is CrMnFeCoNi and its manganese-free derivative CrFeCoNi, both forming single-phase fcc structures (Ref 10). The influence of various additional alloying elements has been investigated. The addition of aluminum clearly affects the phase formation, microstructure, and properties. For the equimolar alloy AlCrFeCoNi, a single-phase bcc structure is formed in the as-cast condition (Ref 11). The investigation of alternative production routes and heat treatments has revealed the formation of an additional fcc and tetragonal σ-phase depending on the manufacturing conditions (Ref 12, 13). Furthermore, detailed investigations on sintered and cast AlCrFeCoNi alloys detected the presence of a disordered bcc phase, in addition to the chemically ordered bcc (B2) phase (Ref 14, 15). The phase formation in dependence of the production route and heat treatment distinctly influences the hardness and wear resistance (Ref 16, 17).

Apart from microstructure, phase formation, and mechanical properties, the corrosion behavior of the alloy system AlCrFeCoNi and other HEAs has also been investigated. The corrosion resistance of cast, sintered, and additively manufactured HEAs in NaCl and H_2_SO_4_ electrolytes has been assessed (Ref 18, 19). Investigation of spark plasma sintered AlCrFeCoNi in 0.5 M H_2_SO_4_ by Zhou et al. revealed distinct passivation. Subsequent annealing was found to impair the corrosion resistance due to the formation of additional phases and hence a more heterogeneous condition (Ref 20). Detailed investigations by Parakh et al. on AlCrFeCoNi showed a distinct influence of the phase fraction (bcc and fcc phases), grain size, and dislocation density on the pitting resistance as tested in 3.5 wt.% NaCl electrolyte (Ref 21). Thermally sprayed AlCrFeCoNi coatings produced by atmospheric plasma spraying (APS) were investigated by Mu et al. in 3.5 wt.% NaCl electrolyte, revealing a high corrosion resistance. The formation of a passive film, which is comprised of Cr and Fe oxides, could be proven despite the relatively heterogeneous condition due to feedstock oxidation during thermal spraying (Ref 22). Investigations of laser cladded coatings in 0.6 M NaCl electrolyte also clearly showed passivation and an improved corrosion resistance in comparison to the stainless steel AISI 304L (EN 1.4306) (Ref 23).

Due to the promising reported corrosion properties as mentioned above, the alloy AlCrFeCoNi was selected for this study. The high-kinetic energy processes of HVOF and HVAF thermal spraying have been selected to enable the deposition of dense coatings with low in situ oxidation during spraying. The influence of the coating process on the formed microstructure and resulting properties was investigated. Furthermore, detailed studies to determine resistance to pitting and passivation behavior have been conducted using NaCl and H_2_SO_4_ electrolytes.

## Materials and Methods

Feedstock powder of the equimolar alloy was produced by NANOVAL GmbH & Co. KG (Berlin, Germany) using inert gas atomization and air classification. For the HVOF thermal spray process, the fraction of −45 +15 µm was used, whereas a fraction of −33 +15 µm was used for the HVAF coating process. The resulting particle size distribution was measured by laser diffraction analyses using a Cilas 920 device (Cilas, Orléans, France). Mild steel substrates (EN 1.0045) with ∅40 mm and 6 mm thickness were prepared by grit blasting prior to coating using Alodur EK F 24 blasting media (−850 +650 µm) with a pressure of 2.5 bar under an angle of 70°. The HVOF coatings were deposited by a K2 liquid-fueled system (GTV Verschleißschutz GmbH, Luckenbach, Germany), employing the parameters summarized in Table 1.

**Table 1:** HVOF coating parameters for AlCrFeCoNi

O_2_ (l/min)	850
Kerosene (l/h)	22.5
λ	1.1
Carrier gas (Ar) flow (l/min)	2 × 11
Nozzle	100/12
Powder feed rate (g/min)	2 × 35
Spraying distance (mm)	360
Surface speed (m/min)	60
Spray-path offset (mm)	5
Coating layers	15

The HEA AlCrFeCoNi powder was also sprayed using a HVAF coating process. For this purpose, a M3^TM^-HVAF spray system (Uniquecoat, Oilville, VA, the USA) was employed. Mild steel substrate specimens in the form of ∅25 mm round coupons of 6 mm thickness were mounted on a horizontal rotating fixture and sprayed with parameters summarized in Table 2. As depicted in the table, a nozzle designated 4L4C (having 250 mm length and 22.75 mm exit diameter) was employed, while a short injector was used to introduce the HEA powder axially into the air-fuel jet.

**Table 2:** HVAF coating parameters for AlCrFeCoNi

Air pressure (psi)	110
Fuel 1 (Propane) pressure (psi)	100
Fuel 2 (Propane) pressure (psi)	105
Carrier gas (N_2_) flow (l/min)	40
Nozzle	4L4C
Powder feed rate (g/min)	100
Spraying distance (mm)	300
Surface speed (m/min)	100
Spray-path offset (mm/rev)	5
Coating layers	23

Cross-sections of the feedstock and coatings were prepared by standard metallographic procedures and investigated by scanning electron microscopy (SEM) using a LEO 1455VP microscope (Zeiss, Jena, Germany) operated with an acceleration voltage of 25 kV. For visualization of material contrast, a backscattered electron (BSE) detector was used. The chemical composition was determined by coupled energy-dispersive x-ray spectroscopy (EDS) using a calibrated EDS GENESIS (EDAX, Mahwah, NJ, the USA) system for standardless quantitative measurements. Therefore, area analyses at a magnification of 500x have been performed. For both coating types, a measurement area of approximately 40,000 µm^2^ was investigated. At least three measurements have been conducted to determine the average value. For the conducted measurements, a standard deviation in the range of ± 0,1−0,2 at.% occurred. Furthermore, the porosity was determined by optical microscopy image analyses using a GX51 microscope (Olympus, Shinjuku, Japan) and the Stream software (Olympus, Shinjuku, Japan). Ten images, which were taken with a magnification of 500x, have been considered to determine the average and standard deviation.

X-ray diffraction (XRD) measurements were conducted to determine the phases formed using a D8 Discover diffractometer (Bruker, Billerica, MA, the USA). For all investigations, a diffraction angle range (2θ) of 20° to 130° was considered using Co Kα radiation. The roughness (*R*_*a*_, *R*_*z*_) of the coatings in the as-sprayed condition was determined by tactile measurements using a Hommel-Etamic T8000 device (Jenoptik, Villingen-Schwenningen, Germany) according to ISO 4288 (Ref 24). Across the coating cross-sections, the coating microhardness value (HV 0.1) was determined with a Wilson Tukon 1102 device (Buehler, Uzwil, Switzerland). Ten measurements have been considered to calculate the average value and standard deviation.

Prior to the corrosion investigations, the surface of the coatings was ground down to Grit 4000 (US#1200). For the investigation of pitting resistance and passivation behavior, potentiodynamic polarization curves were recorded in 0.5 M NaCl and 0.05 M H_2_SO_4_ aerated electrolytes. A circular sample surface with a diameter of 10 mm was investigated. For all measurements, a three-electrode arrangement with a Pt sheet (2 × 2 cm^2^) as a counter electrode was used. A reference electrode Ag/AgCl (saturated KCl) as well as a Haber-Luggin capillary was applied. The tests were conducted at room temperature with a PS6 potentiometer (Sensortechnik Meinsberg, Meinsberg, Germany). At least three measurements were made for each coating and test condition. The test parameters are summarized in Table 3.

**Table 3:** Test parameters of the potentiodynamic polarization measurements

	Corrosion	Passivation
Electrolyte	0.5 M NaCl	0.05 M H_2_SO_4_
Potential scan	−250 to +700 mV 0.1 mV/s	−1000 to +1800 mV 0.5 mV/s

The corrosion potential (*E*_corr_) and the corrosion current density (*i*_corr_) were determined by fitting the Butler–Volmer equation in the proximity of *E*_corr_. Therefore, a range of approximately ± 20 mV around *E*_corr_ has been considered. For the investigation of the predominant corrosion mechanisms, potentiodynamic polarization measurements were conducted and interrupted at a defined current density of 2 mA/cm^2^ to enable direct comparability. The surface of the samples was investigated with a VHX 500 digital microscope (Keyence, Osaka, Japan) to evaluate the corrosion mechanisms.

## Results and Discussion

### Feedstock Characterization

**Fig. 1:**
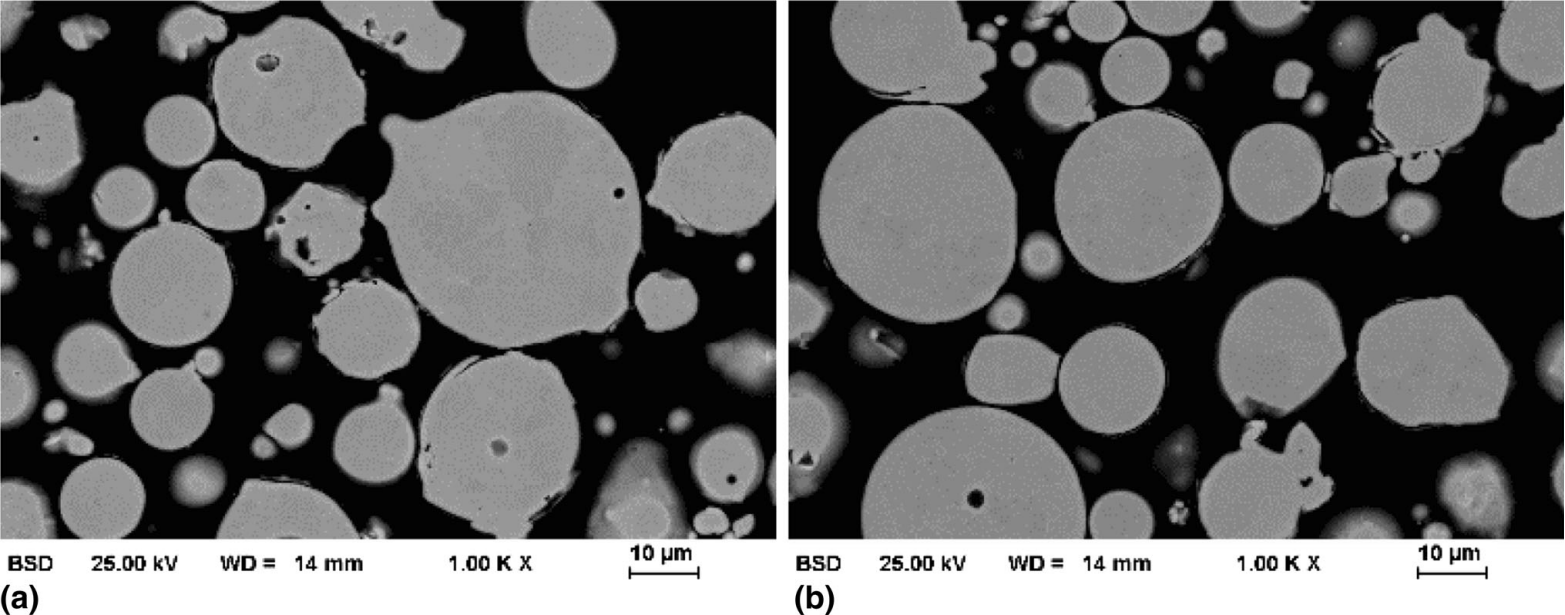
SEM images (BSE) of gas-atomized AlCrFeCoNi powder: (a) HVOF feedstock -45 +15 µm and (b) HVAF feedstock -33 +15 µm.

The morphology and microstructure of the feedstock powders were investigated. Typical images of particle cross-sections obtained using a BSE detector for material contrast visualization are shown in Fig. 1.

A predominantly spherical morphology can be observed for the feedstock, which is typical for gas-atomized powder. Within bigger particles, a dendritic structure is formed. However, no distinct material contrast occurs, which indicates that there is no deviation of the local chemical composition. The particle size distribution was determined to be in the range of −42 +14 µm for the HVOF feedstock. A range of −38 +13 µm was measured for the HVAF feedstock.

### Coating Characterization

**Fig. 2:**
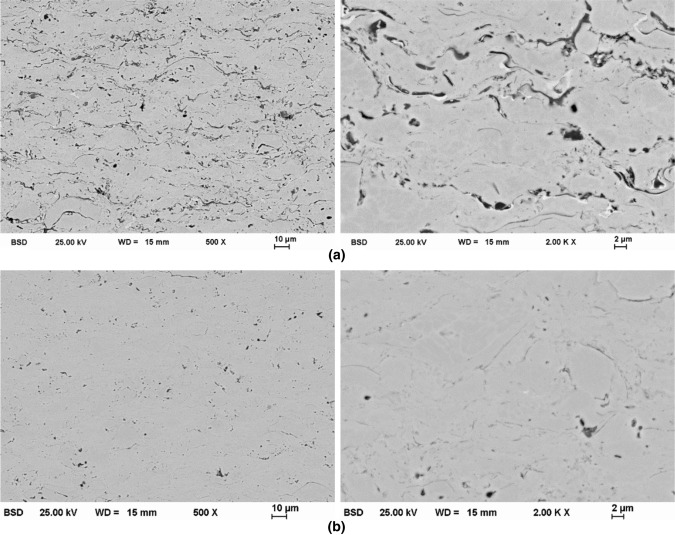
Microstructure (BSE) of AlCrFeCoNi coatings produced by: (a) HVOF and (b) HVAF thermal spraying.

The cross-section microstructures of the coatings were investigated by SEM. Typical microstructures are shown in Fig. 2. At low magnification, a lamellar structure can be observed in the AlCrFeCoNi coating produced by HVOF. Oxide lamellae and pores are partially formed between the spray particles. Image analyses revealed a porosity of 0.6 ± 0.2%. In accordance with the feedstock powder, a dendritic structure can be observed at higher magnification within single spray particles. However, no distinct material contrast occurs. For the coatings produced by HVAF, no distinct oxide lamellae are formed due to the reduced thermal input in the coating process and the corresponding suppression of oxygen pick-up during spraying. In addition, the porosity is reduced in comparison to the HVOF coatings to a value of 0.3 ± 0.1%. At higher magnification, the dendritic structure of the feedstock powder particles can be observed.

**Fig. 3:**
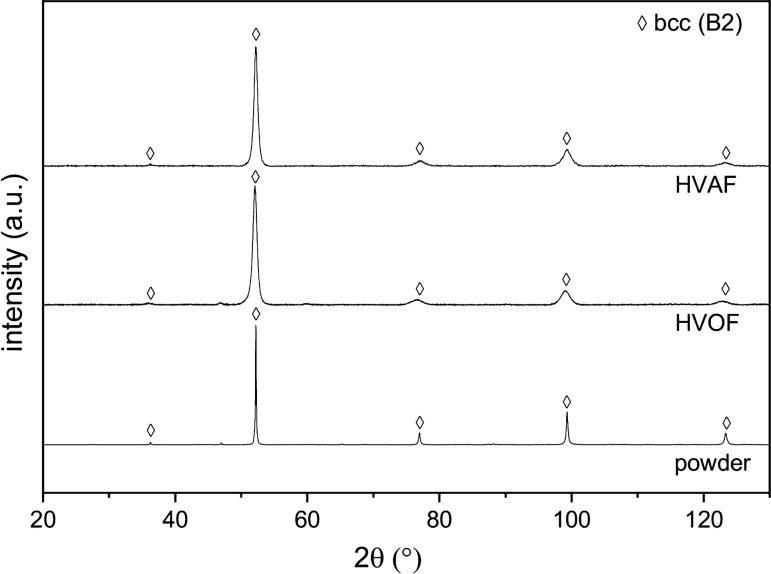
Diffractograms of AlCrFeCoNi feedstock and coatings.

The chemical composition of the powder and coatings was measured by EDS. The results are summarized in Table 4. In comparison to the nominal composition, no significant deviation appears to occur during the atomization to produce the feedstock powder. The processing by HVOF and HVAF thermal spraying also causes no significant changes in composition. The phase constitution of the feedstock and coatings was analyzed by XRD. The resulting diffractograms are shown in Fig. 3. The diffraction peaks of the atomized powder can be assigned to a bcc phase. The superstructure peak at a diffraction angle of 36.3° ({100} peak) indicates the formation of a chemically ordered B2 structure. The formation of this primary phase is in accordance with previous investigations on arc-melted alloys of the same composition, which revealed the formation of a single-phase bcc (B2) structure as well (Ref 11). The cooling conditions in arc-melting and atomization processes suppress the formation of additional phases, which have been detected for alternative production routes and additionally heat-treated material (Ref 12, 13). The processing of the feedstock by HVOF or HVAF thermal spraying also causes no changes in the phase composition. A bcc (B2) phase has been determined in all conditions. The evaluation of the lattice parameter revealed a value of *a* = 2.87 Å for all states. No influence of the production route on the lattice parameter could be observed due to the low thermal input of both coating processes.

**Table 4:** Nominal and measured chemical composition of the feedstock powder and coatings, in at.%

	Al	Cr	Fe	Co	Ni
*Nominal*	*20.0*	*20.0*	*20.0*	*20.0*	*20.0*
powder	20.0	19.3	20.9	18.8	21.1
HVOF	19.8	18.8	20.9	19.0	21.5
HVAF	20.2	20.6	20.7	19.7	18.9

**Fig. 4:**
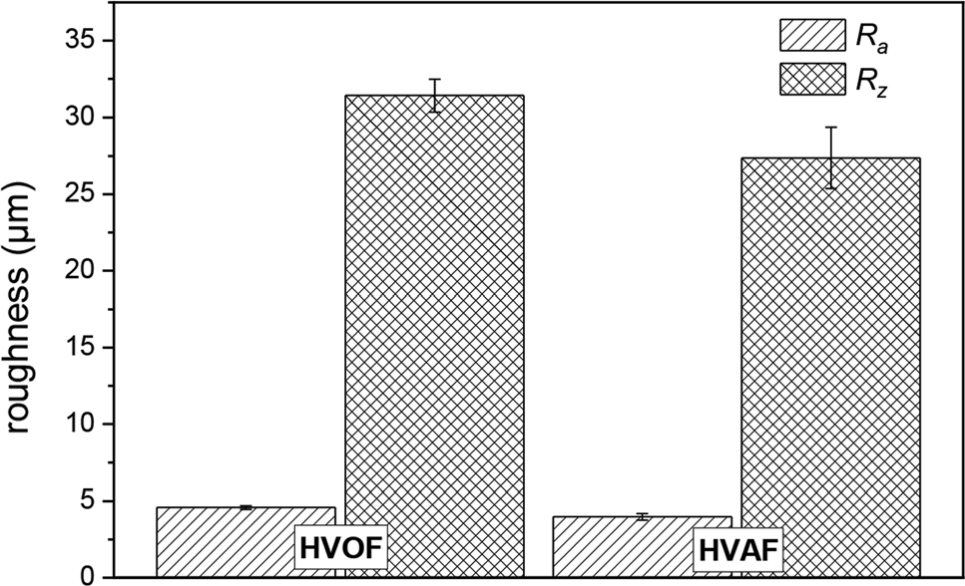
Roughness values of AlCrFeCoNi coatings produced by HVOF and HVAF thermal spraying.

The influence of the production route on the resulting surface properties was investigated. The roughness values of the coatings are summarized in Fig. 4. Roughness values of *R*_*a*_: 4.6 ± 0.1 µm and *R*_*z*_: 31.4 ± 1.1 µm have been determined for the HVOF coating, whereas processing of the feedstock with reduced particle size by HVAF results in decreased roughness values of *R*_*a*_: 4.0 ± 0.2 µm and *R*_*z*_: 27.4 ± 2.0 µm. Therefore, the effort for mechanical finishing of the coatings can be reduced.

Microhardness measurements revealed values of 600 ± 50 HV 0.1 for the HVOF coatings. For the coatings produced by HVAF, an increased microhardness of 680 ± 20 HV 0.1 was determined. This behavior might be caused by the finer powder fraction, resulting in a decreased grain size. Furthermore, the standard deviation is reduced, indicating the formation of a more homogeneous coating in the case of HVAF. This further corroborates the results from the microstructural investigations discussed above, where a lower content of oxide lamellae and porosity is present.

### Corrosion Behavior

**Fig. 5:**
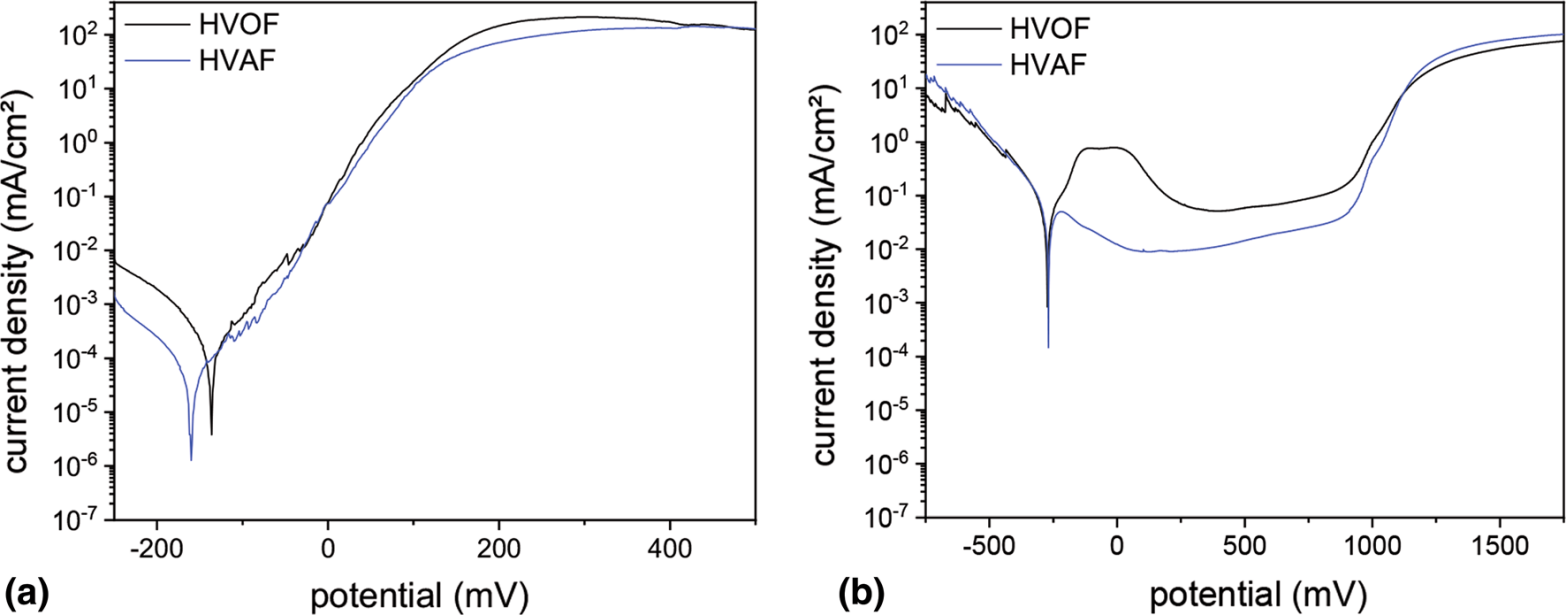
Potentiodynamic polarization curves of AlCrFeCoNi HVOF and HVAF coatings measured in: (a) 0.5 M NaCl and (b) 0.05 M H_2_SO_4_ electrolyte.

The corrosion and passivation behavior was investigated by potentiodynamic polarization tests using NaCl and H_2_SO_4_ electrolytes. Typical potentiodynamic polarization curves are shown in Fig. 5. No passivation of both coating types can be observed for the measurements in 0.5 M NaCl electrolyte. In contrast, a distinct passivation occurs for the measurements in 0.05 M H_2_SO_4_ electrolyte.

**Fig. 6:**
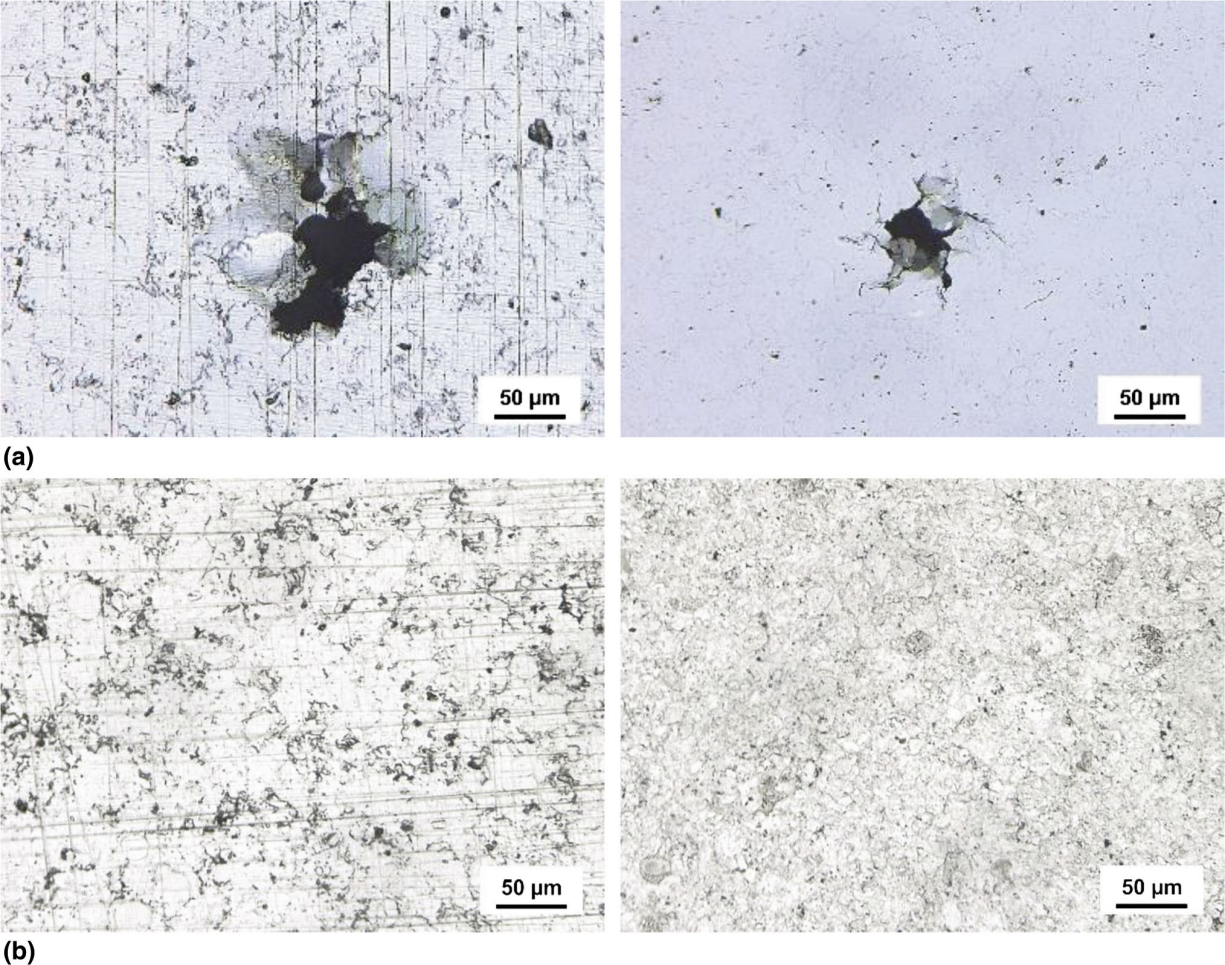
Surface of AlCrFeCoNi HVOF (left) and HVAF (right) coatings after the corrosion attack, interrupted at a current density of 2 mA/cm^2^ in: (a) 0.5 M NaCl electrolyte and (b) 0.05 M H_2_SO_4_ electrolyte.

For investigations to gain insight into the corrosion mechanisms, the surface of the specimens was examined after they were subjected to corrosion testing, which has been interrupted at a current density of 2 mA/cm^2^. Light microscopic images of the sample surface are shown in Fig. 6. For both coating types tested in NaCl electrolyte pitting corrosion occurs at low current densities. Cracks and localized corrosion attack can be observed. However, investigation of cross-sections reveals that these are limited to the surface. For low current densities, no penetration of the electrolyte or delamination of the coating could be observed. With increasing current density, extensive delamination and coating failure occurred. In contrast to previous investigations on APS coatings of the same composition, there is no passivation for the investigated test conditions (Ref 22).

Light microscopic images of the sample surface after corrosion investigation in H_2_SO_4_ electrolyte are shown in Fig. 6(b). Both coating types revealed no distinct corrosion reactions, although the specimens were subjected to corrosion well past the passive region. For specimens investigated in the full potential range (−1000 to +1800 mV) in H_2_SO_4_, neither severe corrosion attack nor delamination could be observed. Only a thin layer at the surface is affected. Previous potentiodynamic investigation of the spark plasma sintered HEA AlCrFeCoNi in H_2_SO_4_ electrolyte revealed a distinct passivation, although a multiphase microstructure was formed due to the different production conditions (Ref 20). The average characteristic values for both corrosion types are summarized in Table 5.

**Table 5:** Characteristic values of potentiodynamic polarization measurements in NaCl and H_2_SO_4_ electrolyte for AlCrFeCoNi HVOF and HVAF coatings

Coating	Electrolyte	*E*_corr_ (mV)	*i*_corr_ (mA/cm^2^)
HVOF	NaCl	−133 ± 26	(1.5 ± 1.0) × 10^−4^
HVAF	NaCl	−155 ± 13	(0.7 ± 0.4) × 10^−4^
HVOF	H_2_SO_4_	−272 ± 19	(3.0 ± 1.7) × 10^−2^
HVAF	H_2_SO_4_	−260 ± 19	(2.1 ± 0.7) × 10^−2^

The comparison of both coating types tested in NaCl electrolyte reveals a slightly nobler *E*_corr_ for the HVOF coating. However, *i*_corr_ is reduced by a factor of two for the HVAF coating. The investigation of the passivation behavior in H_2_SO_4_ electrolyte reveals distinct differences between both coating types. While *E*_corr_ is not significantly affected by the coating type, *i*_corr_ is distinctly reduced for the HVAF coating. Furthermore, the passivation current density is reduced as well. In the passive region, the HVAF coatings show a lower mean current density (0.013 vs. 0.073 mA/cm^2^) compared to the HVOF coatings, which indicates the formation of a denser and more protective passive layer. For both coating types, a slight increase in current density with increasing potential can be observed in the passive region. This might be caused by the roughening of the surface due to the corrosion process, which results in a larger surface and hence increased current. The pitting potential is not significantly influenced by the coating process (947 vs. 957 mV), which seems to be determined by the chemistry of the coating.

## Summary and Conclusions

Inert gas-atomized feedstock of the HEA AlCrFeCoNi was successfully processed by HVOF and HVAF thermal spraying. Due to the low thermal input, the bcc (B2) structure can be retained in both coating types. The content of pores and oxides is reduced in the HVAF coatings. Furthermore, the roughness of the HVAF coatings in the as-sprayed condition is reduced in comparison to the HVOF coatings. Hardness measurements revealed an increased hardness for the HVAF coatings, which is also affected by the processing of feedstock with a reduced particle size.

Detailed investigations of the corrosion behavior have been conducted to determine the resistance against pitting and the passivation behavior. Therefore, potentiodynamic polarization tests in NaCl and H_2_SO_4_ electrolyte have been conducted. No passivation was observed in both coating types when tested in NaCl electrolyte. However, the corrosion current density is distinctly reduced for the HVAF coatings.

Investigation of the passivation behavior in H_2_SO_4_ electrolyte also reveals significant differences between both coating types. While a passive region can be observed in both coating types, the passivation current density and the current density in the passive region are distinctly reduced in case of the HVAF coating. This indicates an improved passivation behavior and the formation of a denser and more protective passive layer.

The coating process of HVAF thermal spraying shows a high potential for the processing of HEAs, enabling the production of coatings with reduced structural defects and increased hardness. Due to the high cooling speeds in the atomization process, a metastable single-phase state is adjusted for the equimolar AlCrFeCoNi powder. This state is retained for the HVOF and HAVF coatings because of the low thermal input in the coating process. In comparison with coatings produced by HVOF thermal spraying, the corrosion resistance in both NaCl and H_2_SO_4_ electrolytes is improved for the HVAF coatings.
